# Secure Cross-Layer Mobile Sensing Framework for Real-Time Disaster Reporting and Visualisation Using a Mobile Application

**DOI:** 10.3390/s25216766

**Published:** 2025-11-05

**Authors:** Rashid Mustafa, Jun Han, Nurul I. Sarkar, Krassie Petrova

**Affiliations:** 1 School of Information Technology, Whitecliffe College of Arts and Science, Auckland 1010, New Zealand; rashidm@whitecliffe.ac.nz (R.M.); junh@whitecliffe.ac.nz (J.H.); 2 Department of Computer and Information Sciences, Auckland University of Technology, Auckland 1010, New Zealand; krassie.petrova@aut.ac.nz

**Keywords:** disaster management, cross-layer architecture, mobile applications, user-event reporting, hazard visualization, security implementation

## Abstract

As the number of natural and man-made catastrophes has increased in recent years, there has been an increasing need for quicker and more efficient disaster response. Information from traditional sources, such as radio, television, and websites, is sometimes incomplete or delayed. While mobile applications provide a means of enhancing real-time crisis communication, a secure mobile app-based solution has not been fully explored yet. In this paper, we propose a secure and scalable cross-layer disaster management system architecture. To validate the system performance, we developed a user-centred, scalable mobile application known as the disaster emergency events application (DEAPP) for real-time disaster reporting and visualization including disaster notifications and observing the affected areas on an interactive map. The solution connects a web-based backend, cloud database, and native Android mobile app via a cross-layer architecture. Role-based access control, HTTPS connection, and verified event publication all contribute to security. Moreover, Redis caching is employed to expedite data access in emergency situations. The need to verify publicly filed reports to prevent false alarms, safeguard real-time data transfer without slowing down the system, and create an intuitive user interface for individuals in high-stress circumstances are some of the issues that the project attempts to solve. The results obtained show that a mobile system that is secure, scalable, and easy to use can enhance catastrophe awareness and facilitate quicker emergency responses. For developers, researchers, and emergency organisations looking to leverage mobile technology for disaster preparedness, the findings provide helpful insights.

## 1. Introduction

The frequency, scale, and complexity of disruptive events that endanger lives, infrastructure, and economic activity have intensified, spanning floods, wildfires, earthquakes, industrial accidents, and public-health emergencies. At the same time, mobile technologies and data-driven platforms have become central to preparedness, warning, and coordination, yet many deployed solutions still rely on one-way alerting models with limited interactivity, delayed situational updates, or static user settings. Systematic reviews and domain applications point to both their promise and persistent gaps: crowdsourced hazard apps emphasise notifications and engagement but vary in verification and usability [1]; evacuation support tools demonstrate the value of real-time routing but depend on model accuracy and timely delivery [2]; IoT/edge/cloud pipelines achieve low-latency sensing and scalable dissemination [3]; and trustworthy-AI studies stress explainability, bias control, and data-fusion challenges in multi-hazard settings [4]. Broader work on digital transformation in disaster management similarly highlights fragmented implementations and the need for integrated, interoperable architectures [5].

Security and resilience remain cross-cutting concerns. Cross-layer defences at physical/MAC/network levels can improve delivery ratios and energy efficiency under adversarial conditions [6], while SDN/NFV approaches help isolate threats and preserve quality of service across slices in next-generation networks [7]. From a user-centric standpoint, purpose-built platforms for medically vulnerable populations show how structured data flows and administrative workflows can strengthen preparedness [8]; emergency medical coordination systems demonstrate secure data exchange and location-aware operations [9]; and web-based 3D GIS underscores the value of interactive visualisation and integrated decision support [10]. Community-facing mobile/web solutions illustrate how shared location, alert orchestration, and resource tracking can elevate situational awareness [11]. Complementary paradigms—crowdsensed early-warning via smartphone sensors [12], handset-derived advanced mobile location to PSAPs [13], and bystander-activation for CPR with AED navigation [14]—further motivate designs that couple reliability with human-centred workflows. A second stream of the digital-transformation literature converges on the same conclusion: piecemeal tools should evolve toward coherent, scalable, and secure ecosystems [5]. In this study, a cross-layer architecture is adopted to enable coordinated optimization across the application, network, and data layers. Unlike traditional isolated designs, the cross-layer approach promotes vertical interaction between these layers, allowing for adaptive feedback and resource adjustment in real time. Such integration is essential in disaster scenarios characterized by high concurrency, strict security requirements, and low user-operation tolerance, ensuring reliability even under unstable network conditions.

Emerging architectures for post-disaster connectivity and data integrity—e.g., blockchain-enabled UAV coordination and tamper-resistant exchanges [15,16], offline/multilingual voice networks backed by decentralised ledgers [17], and cross-layer resilience assessment across application/infrastructure [18]—reinforce two design imperatives for public-facing systems: (i) robust end-to-end security with auditable provenance, and (ii) low-friction interfaces that minimise cognitive load in high-stress contexts. In parallel, humanitarian open-source risk-monitoring platforms [19] and global innovation mappings [20] document wide adoption of AR/VR, IoT, UAVs, and mobile apps in preparedness and response. Mobile real-time data-aggregation pipelines show that near-instant reporting and scalable ingestion are feasible in practice [21], while corporate-level assessments of mobile emergency apps expose recurrent weaknesses in encryption, API hardening, and data handling—underscoring the necessity of rigorous security baselines [22]. Visualization research for emergency training also emphasises clarity, cognitive economy, and integration constraints that are directly relevant to on-device hazard mapping and alert comprehension [23].

Taken together, the literature indicates a clear opportunity: to pair citizen-driven, two-way reporting with verified publication and intuitive visualisation, inside a secure cross-layer architecture that sustains performance under load. This paper pursues that opportunity by designing and evaluating a mobile system that supports dynamic incident submission, administrative verification, and real-time map-based visualisation—while enforcing security and usability constraints drawn from the foregoing evidence.

### 1.1. Research Challenges

Developing a secure, responsive, and user-oriented mobile framework for real-time disaster reporting poses several persistent obstacles that cut across architectural, operational, and human factors. This study identifies three overarching challenges that define the research space:

1. Balancing Security with Performance Across Layers: Maintaining confidentiality, integrity, and availability between mobile clients, web servers, and cloud databases is difficult when system load spikes or connectivity drops. Traditional architectures often privilege speed at the expense of protection. The challenge, therefore, lies in designing a cross-layer structure that preserves throughput and responsiveness while sustaining robust encryption, role-based authentication, and resilient caching mechanisms.
2. Ensuring Reliability of Public-Sourced Information: Crowdsourced disaster reports can drastically improve situational awareness but also introduce inaccuracies, duplication, or intentional false alarms. Without rigorous vetting, such misinformation can undermine emergency coordination. A key challenge is constructing a validation framework that verifies user submissions before publication—preserving openness while ensuring authenticity.
3. Designing for Cognitive Clarity in High-Stress Environments: During emergencies, users face time pressure, stress, and inconsistent network access. Complex or text-heavy interfaces impede action when simplicity is critical. The third challenge, therefore, concerns designing an interaction model that is intuitive, accessible, and effective for all users—even those with limited technical literacy—under unpredictable conditions.

These challenges collectively motivate the need for a resilient cross-layer system that remains secure, credible, and usable in real-world disaster scenarios.

### 1.2. Research Questions

A practical mobile system for real-time disaster reporting and visualisation must (i) safeguard confidentiality, integrity, and availability across the client–server–database path; (ii) validate publicly submitted reports to limit misinformation without sacrificing timeliness; and (iii) reduce cognitive effort so non-expert users can act quickly under stress [1,2,3,4,5,6,7,8,9,10,11,12,13,14,15,16,17,18,19,20,21,22,23]. In this study, we address the following three research questions:

In response to these challenges, this study formulates three focused research questions that guide the design and empirical evaluation of the Disaster Emergency Events Application (DEAPP):

- **RQ1.** What secure cross-layer architecture can sustain real-time disaster reporting and danger-zone visualisation without compromising system performance? This question investigates how an integrated Android–Spring Boot–MySQL framework, supported by HTTPS protocols and Redis caching, can deliver end-to-end protection and responsiveness under varying network loads.
- **RQ2.** What mechanisms can validate public disaster reports while minimising the risk of false or misleading information during emergencies? This question examines the effectiveness of an administrator-verification workflow and explores potential extensions using blockchain provenance or AI-assisted anomaly detection to maintain data integrity when user volumes surge.
- **RQ3.** What user-interface and system-design principles ensure accessibility, ease of use, and effective information delivery under high-stress conditions? This question evaluates whether streamlined forms, automatic location pinning, and integrated news feeds reduce cognitive load and enable rapid, accurate participation from non-expert users.

Together, these questions transform the domain-wide obstacles identified above into actionable research directions that define the technical and human-centred contributions of this work.

### 1.3. Study Contribution

We developed and evaluated the Disaster Emergency Events Application (DEAPP), a mobile system that: (i) accepts dynamic, GPS-assisted public incident reports, (ii) applies an administrative verification workflow prior to publication, and (iii) renders affected zones on an interactive map in real time. The implementation adopts a layered design with a native Android client, a Spring Boot administrative backend, and a MySQL datastore, communicating via HTTPS; performance under contention is sustained with in-memory caching for frequently accessed artefacts (e.g., event summaries and map layers). The design choices are informed by the reviewed evidence on crowdsourcing and engagement [1,11], low-latency sensing and dissemination [3,21], secure and resilient networking [6,7,22], and effective visualisation for rapid comprehension [10,23]. The resulting contribution is a secure, performance-aware, and usability-focused framework for citizen-driven disaster reporting and visualisation that can strengthen community resilience and accelerate reliable information flows during crises.

The main contributions of this paper are summarised as follows:

- End-to-end secure cross-layer architecture for real-time disaster intelligence: We develop and validate a layered design (Android ↔ Spring Boot ↔ MySQL) with role-based access, HTTPS APIs, and in-memory caching to sustain responsiveness under surge while preserving confidentiality, integrity, and availability.
- Verified two-way reporting pipeline that limits misinformation: To this end, we develop a mobile application called Disaster Emergency Events Application (DEAPP) for real-time disaster reporting and visualisation. The civil defence news integration and official updates are also included in DEAPP. We introduce a governance workflow where public submissions default to review and are released only after administrative verification; this process supports rapid broadcast once approved and is designed for extensibility (e.g., automated or provenance-aided checks).
- Usability-first interaction model for high-stress contexts: We develop mobile user interfaces to minimise cognitive load via automatic location pinning, a short event form (mandatory name and timestamp), and an integrated civil-defence news feed, enabling non-experts to act quickly during emergencies.
- Generalizable blueprint for secure, performance-aware, citizen-driven visualisation: To this end, we develop secure packet-based communication between a mobile client and an HTTPS Server. By integrating verified crowdsourced reports with responsive map layers and hardened networking practices, the work contributes a reusable framework for mobile disaster systems that strengthens community resilience and accelerates trustworthy information flows.

## 2. Related Work

Evaluations of 77 apps from the Google Play Store highlight the value of combining user-driven features, AI, IoT, and crowdsourcing. Applications for managing disasters on mobile devices, especially those pertaining to flood preparedness, are essential for communication, alerting, and coordinating operations. Their ability to improve community resilience over the long term hinges on gamified engagement, inter-agency cooperation, timely updates, and interactive communication skills [1]. Mobile applications like EscapeWildFire demonstrate strong potential for real-time evacuation support during wildfires, validated through case studies in Cyprus and a historic Texas fire. Their effectiveness, however, hinges on precise wildfire modelling, rapid notification, and continuous system improvements, with open-source availability encouraging global adoption by fire authorities [2]. To enable secure emergency response, Zhang et al. introduce and evaluate an Internet of Things (IoT)-based system that integrates cloud platforms, edge nodes, and heterogeneous sensors, low-latency notifications in a variety of situations, such as medical emergencies, gas leaks, fires, and accidents [3]. Its exceptional accuracy, dependability, and scalability are highlighted by comparison with cutting-edge solutions, highlighting its potential for implementation in smart cities, industries, and infrastructure with upcoming AI-driven improvements. Trustworthy AI is increasingly recognised as an important tool in natural disaster management. Methods such as explainable AI (XAI), machine learning, deep learning, data fusion, and multi-criteria decision-making can support predictive models, early warning systems, and resource allocation. While highlighting its potential and difficulties—such as explainability, bias, ethics, and data limitations—a systematic evaluation of 108 studies also provides important insights and answers to help shape future disaster resilience plans [4]. A theoretical review methodology is used by Fischer-Preßler et al. [5] to describe the state of digital transformation (DT) in disaster management (DM), making a distinction between organizational initiatives that are facilitated by IT and more general digital initiatives. It describes future research directions in DM, highlights differences from industrial DT, and unifies disparate studies by offering an integrative framework.

This study integrates the physical, MAC, and network layers in a cross-layer security system designed to detect and isolate wormhole and blackhole attacks in wireless ad hoc networks. The system uses an Enhanced Support Vector Machine (E-SVM) with NS3 simulation and shows improvements in energy efficiency, packet delivery ratio, and QoS, while remaining protocol-independent and reducing false positives [6]. Using network function virtualisation (NFV) and software-defined networking (SDN), Allaw et al. [7] offer a cross-layer security (CLS) framework for 5G/6G network slices that guarantees adaptive threat detection, isolation, and resilience. By improving scalability, QoS, and slice integrity through their hybrid SDN/NFV strategy, CLS is positioned as a crucial facilitator for next-generation secure mobile networks. The K-DiPS system, which consists of the web platform K-DiPS Online and the smartphone app K-DiPS Solo, is presented by Nakai et al. [8] It allows medically vulnerable people (MVPs) to enter personal and health information, which is then sent to local governments via the cloud for disaster preparedness and response coordination. Actionable catastrophe training, resource allocation, simulation, and mapping of susceptible persons are all made possible by the system’s smooth MVP-to-government data flows [8]. Perera et al. [9] presented a secure mobile and web-based emergency platform that improves coordination between ambulances and hospitals (EMS) in Sri Lanka by allowing simultaneous AES-256-encrypted health data transmission, real-time GPS tracking of ambulances, and OCR-enabled ID data acquisition. The technology greatly improves the effectiveness and preparedness of emergency response by simplifying interagency communication and protecting patient data. Based on Vue.js and Cesium Digital Earth, Yang et al. [10] create a full web-based interactive 3D GIS for emergency response that includes live spatial analysis, interactive route planning, augmented reality visualisation, landslide susceptibility modelling, and integrated message dispatch. In catastrophe situations, their system is a prime example of how WebGL-powered 3D geospatial tools may improve multi-source data integration, decision support systems (DSS), and situational awareness (SA). By providing real-time location sharing, alert coordination, and resource tracking to promote proactive disaster management, Vera et al. [11] present a mobile (and web) application that improves disaster preparedness and response. Through enhanced emergency response effectiveness, better situational awareness (SA), and smooth data interchange, the platform benefits communities and emergency agencies alike. A crowd-sourced smartphone-based Earthquake Early Warning System (EEWS) that uses accelerometer data to identify seismic events and promptly notify users who may be at risk is provided by the Finazzi et al. [12]. This technology provides essential pre-shaking warnings, improving emergency preparedness through scalable, economical deployment, particularly in areas without conventional seismometers as in Table 1. To improve the accuracy and timeliness of Emergency Services (EMS) responses, the Advanced Mobile Location (AML) system allows smartphones to automatically send handset-derived GNSS/Wi-Fi location data via SMS or HTTPS to Public Safety Answering Points (PSAPs). By enhancing geolocation accuracy to less than 100 m, as standardised by ETSI, AML increases situational awareness (SA) and interoperability among emergency networks [13]. By directing them to nearby AEDs and activating CPR-trained bystanders through GPS-based “CPR Needed” alerts timed with PSAP dispatches, the PulsePoint Foundation [14] improves emergency response through community engagement and increases survival chances. When combined with CAD, radio streaming, and a crowdsourced AED registry, it improves situational awareness (SA) and the Chain of Survival among public safety networks.

Fischer-Preßler et al. [5] review digital transformation (DT) in disaster management and compare IT-based initiatives with broader digital strategies. Their work separates disaster-related digital twin (DT) applications from industrial use cases and summarises existing studies, suggesting possible directions for DT in disaster management (DT-in-DM). To improve UAV fleet security and communication in post-disaster networks (PDNs), Hafeez et al. [15] propose a blockchain-based coordination model. It applies smart contracts, consensus protocols, and distributed ledgers to protect UAV-to-UAV (U2U) communication, making emergency response more scalable and reliable. Similarly, Wang et al. [16] present RescueChain, a blockchain-enabled platform tailored for UAV-assisted disaster rescue (UAV-DR). It combines distributed ledger technology (DLT), edge computing (EC), and artificial intelligence (AI) to achieve low-latency coordination while maintaining trust, efficient resource use, and integrity in post-disaster communication. Behravan et al. [17] explore multilingual crisis communication with a voice-based social network (VSN). The platform uses blockchain for secure, decentralised messaging and AI for speech recognition and translation. Even when normal infrastructure fails, it allows reliable and inclusive communication across communities.

Ramanathan et al. [18] introduce Xaminer, a resilience analysis tool that connects the infrastructure, network, and application layers. Using AI metrics and simulations, it identifies cascading vulnerabilities, improves fault tolerance, and helps strengthen disaster readiness in critical systems. At the global level, the World Food Programme’s PRISM Project (2020–2024) [19] provides an open-source GIS platform that combines satellite imagery, geospatial analysis, and AI forecasting. It enhances early warning systems (EWSs), supports real-time risk assessment, and improves coordination in humanitarian response. Alongside this, the UNDP Innovation Report [20] highlights the role of AR/VR, IoT, UAVs, and digital platforms in disaster management. These technologies have shown benefits in preparedness, rapid response, and recovery, while promoting models of resilience that are scalable, inclusive, and sustainable.

The Real-Time Disaster Information Aggregation Study [21] describes a mobile-based system that combines GPS, cloud computing, and real-time sensing. This design enables fast data collection and reporting, improving situational awareness (SA) and decision-making in critical events. Still, major security issues remain. The Global Mobile Threat Report [22] identifies risks in mobile emergency applications (MEAs), such as weak encryption, unverified APIs, and data leakage. It calls for stronger security standards, compliance with GDPR and HIPAA, and resilience-focused design. Finally, Li et al. [23] evaluate the use of visualisation technologies (VTs) in emergency simulation training (EST). They note how 3D modelling, VR/AR, and GIS platforms can improve realism, decision-making, and situational awareness. However, they also point to limits such as cost, scalability, and data integration, while identifying opportunities for AI- and cloud-based tools. In a related review, Li et al. [23] further examine VT applications in emergency training, outlining current innovations, problems, and directions for future work.

In his review of visualisation technology (VT) in simulation training (ST) for emergencies (EM), Li et al. [23] highlight how it can improve response effectiveness, situational awareness (SA), and decision-making. In order to improve training realism and resilience, the study examines VT tools, techniques, and applications in EM-ST, emphasising innovations, problems, and future directions.

A complete overview is provided in Table 1. Disaster management (DM) has made considerable strides in utilising AI, blockchain, IoT, UAVs, AR/VR, mobile platforms, CLS, and DT to enhance preparedness, response, and recovery, according to the reviewed literature. On the other hand, integration, interoperability, latency, scalability, and holistic security frameworks continue to present difficulties despite advancements in community-driven apps, cross-layer resilience, secure data exchange, and reliable AI. In order to bridge these gaps, our project aims to create a cross-layer architecture that is both secure and energy-efficient for disaster response systems that are powered by mobile devices and the Internet of Things. our architecture will integrate resilience, trust, and real-time communication into a single adaptive framework.

### 2.1. Summary of Related Work

Prior efforts span four recurring strands. First, citizen-facing mobile tools emphasise alerts, engagement and basic crowdsourcing. Reviews of flood-preparedness apps map a rich feature set but show uneven verification and mixed usability under pressure [1]; related community platforms coordinate resources and shared location but depend on administrative curation [11]. A second strand focuses on routing and sensing pipelines for time-critical response: wildfire evacuation support hinges on model quality and delivery speed [2]; IoT/edge/cloud designs demonstrate low-latency, scalable notifications across heterogeneous incidents [3]; and smartphone-based EEWS and handset-derived AML strengthen reach and geolocation in the last mile [12,13,14].

A third strand tackles security, resilience, and governance. Cross-layer approaches aim to improve delivery and energy use under hostile conditions [6] nd to isolate threats in programmable network slices [7]. Blockchain is used in UAV coordination and crisis voice systems to provide tamper resistance, though it also adds integration challenges [15,16,17]. Corporate reviews of emergency applications continue to find problems with encryption, APIs, and data handling, pointing to the need for stronger security standards [22].

Platform and visual analytics research shows both the benefits and costs of situational awareness tools. Web-based 3D GIS and training visualisation improve understanding but remain limited by data fusion and scalability [10,23]. Global initiatives highlight the growing use of mobile, IoT, UAVs, and AR/VR in disaster management [19,20]. Reviews of digital transformation stress the need for consistent, interoperable architectures rather than fragmented solutions [5].

Across these strands, three gaps remain evident: (i) few citizen-reporting frameworks integrate verified information release with comprehensive, cross-layer security resistant to mobile and API-level threats; (ii) performance optimisation—such as contention-aware caching—has seldom been treated as a primary design concern despite the surge traffic typical of emergency events; and (iii) user-interface principles suited to high-stress environments (concise input, automatic location capture, and integration of official data feeds) are inconsistently applied in practice. The proposed DEAPP framework addresses these deficiencies through a multi-tier Android ↔ Spring Boot ↔ MySQL architecture reinforced by HTTPS encryption, role-based controls, and complete auditability; an administrative validation mechanism that authenticates and approves reports prior to publication to prevent misinformation; Redis-supported caching that sustains low-latency interaction; and intuitive mapping and visual tools that reduce user cognitive effort.

This combined emphasis on security, verification, performance, and usability establishes a functional model distinct from earlier single-focus approaches [1,2,3,5,6,7,10,12,13,14,19,22,23]. The summary of related work is presented in Table 2.

### 2.2. Research Gaps

Citizen-facing disaster applications often depend on unfiltered crowdsourced inputs and rely on basic transport-layer encryption, resulting in inconsistent data integrity and uncertain protection across the mobile→API→database chain [1,11]. Two principal weaknesses remain: (i) inadequate review before public dissemination, and (ii) insufficient end-to-end protection covering credential management, role segregation, and cache safety. The proposed framework mitigates these gaps by introducing a controlled validation stage within the administrative workflow, where every submission undergoes authenticated review, logging, and approval through HTTPS-secured APIs, while cached responses remain protected to sustain low-latency performance.

Routing, sensing, and last-mile delivery studies demonstrate timeliness and scale (wildfire evacuation, IoT edge/cloud, smartphone EEWS, AML) but optimise pipelines rather than the human-centred reporting process experience [2,3,12,13,14]. In stressful situations, verbose forms and complex views depress participation and slow comprehension. We explicitly reduce cognitive load through minimum-field submissions with automatic geotagging, verified publication, and a simple hazard-radius visualisation that users can interpret quickly.

Security focused strands cross-layer defences, SDN/NFV slice isolation, and blockchain-based provenance advance resilience [6,7,15,16,17], yet are often network-centric or costly to integrate at municipal scale. Industry assessments further reveal recurring weaknesses in emergency apps (API exposure, crypto misuse, data handling) [22]. Our contribution centres protection where citizen systems operate: RBAC with scoped admin privileges, token/session hygiene, strict input validation with ORM boundaries, and cache layer controls paired with the verification gate to curb misinformation without adding delay.

Work on decision-support platforms and training visualisation improves situational awareness but typically depends on heavy data fusion and analyst-driven workflows [10,23]. Concurrently, humanitarian platforms and digital-transformation syntheses call for interoperable, coherent architectures rather than siloed tools [5,19,20]. We operationalise this at app scale via a modular Android↔Spring Boot↔MySQL stack with clean REST interfaces over HTTPS, Redis-assisted responsiveness, and clear extension points for provenance or automation.

Finally, evaluation practice skews toward simulations (throughput, delivery ratio, model accuracy) and demonstrations; user-facing outcomes under time pressure are less reported. We contribute a user-centred assessment of reporting effort, verification latency, map comprehension, and perceived usability, evidencing that the integrated recipe—verified two-way reporting, application-layer hardening, and surge-aware performance—delivers practical benefits for non-expert users during emergencies.

## 3. Security Implementation in Cross-Layer Architecture

The ability of disaster management systems to visualise hazards and report incidents in real time represents only one dimension of their effectiveness; another is the strength of their underlying security implementation; another is the strength of their underlying security implementation. Cross-layer designs need to protect availability, confidentiality, and integrity for all interacting components, as recent research has shown System Implementation and Security Configuration [7,24]. This calls for an integrated strategy in which the database, backend server, and mobile client are all connected with secure communication protocols, authentication frameworks, caching techniques, and verification workflows. The suggested system complies with best practices found in disaster technology literature, such as blockchain-backed provenance, AI-driven validation, and SDN/NFV-based isolation, by combining HTTPS-secured APIs, role-based access, encrypted token management, and modular backend services. The architecture, therefore, satisfies both technological and user-centric requirements: fending off hostile attacks while guaranteeing reliable information sharing between residents, administrators, and emergency services during stressful catastrophe situations.

### 3.1. System Implementation and Security Configuration

The backend is implemented with Spring Boot 3.3.0 and Spring Data JPA 3.2.5 (Hibernate 6) on Java 17, connecting to MySQL 8.0. This stack enforces parameterised execution paths by default, which prevents query injection and preserves strong transaction reliability (complete, consistent, and recoverable updates).

Minimal configuration (application-properties):

spring.datasource.url=jdbc:mysql://localhost:3306/deapp?useSSL=false
spring.datasource.username=deapp_user
spring.datasource.password=********
spring.datasource.driver-class-name=com.mysql.cj.jdbc.Driver

# JPA/Hibernate
spring.jpa.hibernate.ddl-auto=update
spring.jpa.properties.hibernate.dialect=org.hibernate.dialect.MySQLDialect
spring.jpa.open-in-view=false
spring.jpa.show-sql=false

        Repository Example—EventRepository.java:

@Query("SELECT e FROM Event e
     WHERE e.status = :status AND e.type = :type
     ORDER BY e.createdAt DESC")
List<Event> findByStatusAndType(@Param("status") String status,
                 @Param("type")  String type);

The configuration disables open-session-in-view and relies on named parameters, ensuring that untrusted input is never interpolated into SQL strings.

### 3.2. System Architecture

**Figure 1:**
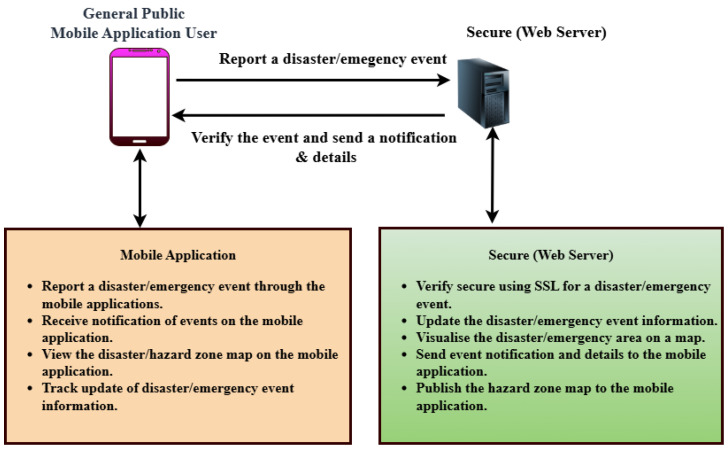
Proposed disaster emergency management system architecture.

When designing a real-time disaster reporting system, the architecture is central to ensuring secure communication, ease of use, and consistent performance. This section outlines the architecture of the Disaster Emergency Events Application (DEAPP). It describes the functional and non-functional requirements and explains the main system flow shown in Figure 1. The diagram shows how users send disaster reports through the mobile app, how these are verified and processed by the web server, and how confirmed information is shared with stakeholders. All communication is secured with HTTPS, and server-side caching is used to speed up responses and improve efficiency.

### 3.3. Functional Requirements

Functional requirements describe the specific capabilities that the system must provide to meet user expectations and achieve its objectives. For the DEAPP prototype, both the mobile application and the web server backend fulfill distinct yet complementary roles.

#### 3.3.1. Mobile Application Functionalities

The mobile application enables the general public to participate in disaster reporting and information access. The key functionalities of the mobile Application are highlighted as follows.

- User Management: Users can register and log in with basic credentials. Once authenticated, they gain access to three core features: New Event, Current Events, and News.
- Event Reporting: By selecting New Event, users can quickly report a disaster using an interactive map with automatic GPS-based location pinning. If necessary, users can adjust the location manually. A simplified form allows entry of essential details, with only the event name and timestamp being mandatory.
- Integration of Mobile Sensors in DEAPP: In addition to the map interface, the DEAPP mobile client directly utilises the smartphone’s built-in GPS position sensor to sense and capture the user’s geographic coordinates (latitude and longitude) during disaster reporting. These sensed spatial data are securely transmitted via HTTPS to the backend server for real-time validation and hazard-zone visualisation. The sensing component ensures that every public report carries verified spatial context, supporting accuracy and reliability in emergency coordination. Future extensions may integrate additional onboard sensors—such as magnetometers, accelerometers, or barometers—to enhance orientation and environmental awareness in field conditions.
- View Disaster Events: The Current Events section displays a list of active disaster events. Selecting an event reveals its details and a hazard zone visualisation on a map.
- Civil Defence News: The News tab provides real-time updates from the New Zealand National Emergency Management Agency, embedded directly into the application for ease of access.

#### 3.3.2. Web Server Functionalities

The web server acts as the administrative control layer:

- Admins can view, edit, or delete user-submitted disaster events. Critical fields such as event severity, coordinates, affected area, and descriptions can be modified as needed.
- All incoming disaster events are initially flagged as “Pending”. The admin must verify each event before it is published to the mobile app. Upon verification, a notification is automatically pushed to users.
- For security, user self-registration is disabled on the backend. Only predefined admin accounts may create or manage other accounts.

This division ensures a secure, structured flow from user input to validated information dissemination.

### 3.4. Non-Functional Requirements

Non-functional requirements address how the system performs rather than what it does. These attributes determine the overall quality, security, and user experience of the prototype.

- The system must support real-time operation and multi-user concurrency. Given that multiple users may report events simultaneously during an emergency, the server must be responsive and capable of handling concurrent requests without delays. Redis caching supports rapid data access, and high-speed internet is assumed for optimal operation.
- The system should be highly available and accessible under all conditions. Disasters may occur at any time, and the platform must be consistently operational to ensure timely information exchange.
- Security is paramount in disaster scenarios. Unauthorised access, denial-of-service attacks, or manipulation of location data could lead to serious consequences. The system adopts HTTPS-secured APIs, Spring Security for token-based authentication, and access control mechanisms to prevent breaches.
- The interface is designed to minimise cognitive load under stress. Auto-location features, concise forms, and embedded news updates enhance usability, allowing users to report incidents and retrieve critical information quickly and efficiently.

### 3.5. System Architecture Overview

**Figure 2:**
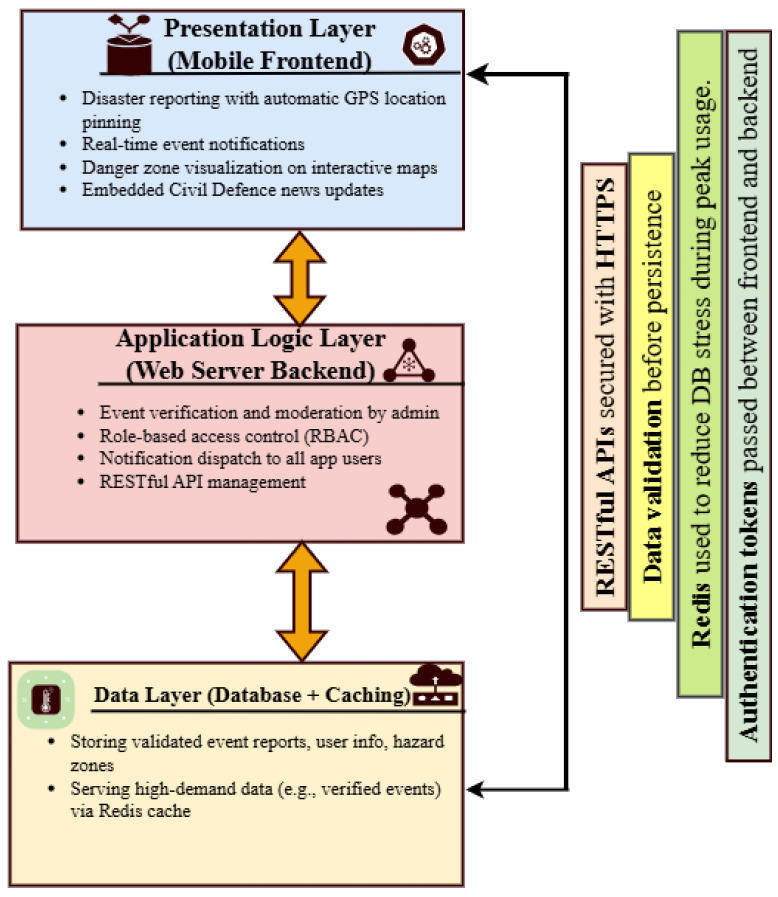
System Architecture Overview of the DEAPP.

The system was developed using a modular, layered architecture, as shown in Figure 2 To guarantee efficient communication, security, and performance during emergency situations, the Disaster Emergency Events Application’s (DEAPP) architecture is essential. The system is divided into discrete functional components that cooperate to facilitate smooth disaster reporting and management, as shown in Figure 2. The underlying database system, the web server backend, and the mobile application are the main parts of the system architecture. The mobile application, which is at the heart of the architecture, enables users to promptly report catastrophic situations by using GPS to tag locations in real time. The backend server receives this data and uses it for management and verification. The server plays a crucial role in processing incoming disaster reports and making sure an administrator verifies their accuracy before making them public. As shown in Table 3, applications such as SyncZone, EscapeWildFire, and K-DiPS integrate user-event reporting, hazard maps, and push-notifications, while security and verification workflows vary significantly across platforms.

Secure protocols like HTTPS are used for communication to safeguard data transfer between the backend and mobile systems. The server uses Redis caching to minimise database access time in order to maximise performance, especially during emergencies when there are heavy loads. Role-based access management is another feature of the backend that makes sure that only authorised users can engage with the system’s essential features, including altering the specifics of a catastrophic occurrence or confirming fresh reports. Rapid distribution of vital disaster-related information to the appropriate stakeholders is made possible by this tiered design, which guarantees the system’s scalability, security, and effectiveness in real-time emergency situations.

### 3.6. AI-Assisted Verification: Data Interfaces and Prototypes

To move beyond a generic mention of “AI verification,” we define a concrete, privacy-aware design comprising (i) a concise evidence payload and (ii) two lightweight background workers that operate off-path to support administrative approvers. Their role is to prioritise incoming submissions for review while maintaining the existing governance sequence from initial intake through verified release.

Evidence payload (HTTPS/JSON): The server normalises inputs and records a compact feature bundle keyed by event_id. Personally identifying data are excluded from model inputs.

POST /api/v1/verify/assist
Content-Type: application/json

{
 "event_id": "e_9f3a1a",
 "reported_at": "2025-08-18T05:41:22Z",
 "coords": {"lat": -36.8485, "lon": 174.7633},
 "event_type": "flood",
 "text": "Road flooded near the bridge.",
 "attachments": [
  {"media_id":"m1","type":"image","sha256":"<hash>"},
  {"media_id":"m2","type":"video","sha256":"<hash>"}
 ],
 "device": {"os":"Android 14","gps_hdop": 2.1},
 "reporter": {"rep":0.72,"prev_reports":5},
 "context": {"rain_mm_2h": 14.6}
}

A background worker retrieves each record, computes the relevant features, and returns a signed assist result. The resulting outputs are stored in Redis for fast retrieval and simultaneously logged in the primary audit store for traceability.

{
 "event_id": "e_9f3a1a",
 "confidence": 0.86,          // 0..1
 "labels": ["geo_plausible","not_duplicate"],
 "rationale": [
  "Within 650 m of active cluster",
  "Rainfall agrees (last 2 h)",
  "Image EXIF time consistent"
 ],
 "version": "assist-0.2.1"
}

Prototype A—Duplicate & Consistency Worker (MinHash + geo-time rules): Clusters near-identical text and checks spatiotemporal plausibility; runs in O(logn) lookup with LSH.

Input: text, coords, reported_at, gps_hdop
Steps:
 1) Shingle text -> MinHash -> LSH index   // near-duplicate candidates
 2) Geo bucket: 500 m grid; Time window: 30 min
 3) Rules:
   - haversine(p, centroid) < R_th
   - |Δt| < T_th AND gps_hdop < H_th
 4) Emit flags {duplicate|unique}, {geo_plausible|implausible} + rationale

Prototype B—Credibility Scorer (shallow, interpretable model). Fast logistic/GBDT over-engineered features; produces a score and top attributions:

Features:
 f1: distance_to_verified_centroid (km)
 f2: time_since_nearby_reports (min)
 f3: reporter_reputation (0..1)
 f4: text_entropy + keyword cues
 f5: gps_hdop
 f6: image_exif_consistency (0/1)
 f7: local_rain_agreement (0/1)

Output:
  confidence in [0,1] + top feature contributions

Integration and safeguards: Results appear in the admin console as approvers retain final control to verify/reject. All assist outputs are versioned and logged (model id, input hash, rationale). If Redis is unavailable, the console gracefully degrades to the baseline queue with no loss of function. This design improves triage speed without altering the security posture or the auditable verification trail.

### 3.7. Implementation Workflow and Data Exchange

Figure 2 presents the secure cross-layer architecture of the Disaster Emergency Events Application (DEAPP). The mobile client layer captures event data through GPS tagging and transmits encrypted JSON payloads via HTTPS. The web server layer (Spring Boot–based) authenticates each request using JWT tokens, validates reports, and caches frequent queries in Redis to maintain sub-two-second response times. Verified information is stored in the MySQL database and disseminated back to users through push notifications and hazard-zone visualisation. Together, these layers ensure confidentiality, scalability, and continuous operation even under high user concurrency. This integrated design forms the backbone of DEAPP’s resilience and real-time performance during disaster events.

### 3.8. Cross-Layer Design Rationale and Performance Analysis

The DEAPP framework is founded on the principle of cross-layer optimisation, where application, network, and persistence tiers cooperate rather than operate in isolation. Such coordination ensures stability when disaster conditions create volatile network loads. At the mobile layer, Android and Material Design were selected to deliver a low-friction interface that supports one-touch reporting, automatic GPS tagging, and offline caching—features that align with low user-operation tolerance during stress. At the server layer, Spring Boot 3.3.0 integrates a reactive request model with Redis caching, providing sub-two-second responses even under high concurrency. Spring Security and JWT authentication strengthen confidentiality and prevent unauthorised access. At the data layer, MySQL 8.0 with Hibernate (JPA 3.2.5) maintains strong transaction reliability and parameterised query protection, ensuring that all database updates are complete, consistent, recoverable, and ensuring consistent data integrity during heavy transactional loads.

High-concurrency testing using Locust 2.31 simulated 100, 500, and 1000 concurrent users. The mean response latency was 1.6 s, with a 95th percentile latency of 2.4 s and a maximum of 3.2 s. Under intermittent-network simulation (30% packet loss), Redis and client-side queues buffered reports for later synchronisation, preventing data loss and maintaining operational continuity. These findings demonstrate that the proposed cross-layer architecture effectively balances scalability, security, and usability in real-world disaster scenarios.

## 4. Secure Packet-Based Communication Between Mobile Client and HTTPS Server

When reporting catastrophic events, confidentiality, integrity, and authenticity are guaranteed by the secure communication between the HTTPS server and the mobile client. Strict certificate validation, including certificate pinning within the mobile application, is enforced for all communications via TLS 1.3 in order to guard against man-in-the-middle attacks. Each request has a distinct nonce and timestamp to guard against replay attacks, and the client authenticates using OAuth2 and JWT bearer tokens. The server uses the idempotency key generated by the mobile application to deduplicate repeated requests, hence reducing duplication in unstable network conditions. The client reports a disaster event, like a “earthquake” or “car crash,” by sending a secure POST request to the server with structured JSON that includes the device data, geographical coordinates, and event details.

Before putting the record in a pending state, the server does schema validation, geospatial sanity checks, timestamp and nonce checks, and JWT validation. In accordance with best practices in cross-layer secure design [7,24], the server acknowledges each event submission with a nonce, unique identifier, and timestamp structured in accordance with ISO 8601 conventions for temporal consistency and interoperability. The acknowledgment that the server sends back includes the latitude and longitude coordinates provided by the device, the precise day, date, and time of reception in ISO 8601 format, the given event number, and the event name. This guarantees that the reporter can instantly verify the information captured by the system, even before administrators have had a chance to verify the incident. Following admin console verification, the event status is changed to “VERIFIED,” and clients get a push notice with the timestamp, location, and confirmed event information. The published record can then be retrieved by users by sending a secure GET request to the server, which gives the authoritative event data, including, if relevant, the hazard radius.

To guard against injection attempts and faulty payloads, the server enforces size restrictions, stringent input validation, refresh methods, and JSON schema tests for short-lived tokens. Token identities, idempotency keys, and request pathways are among the crucial metadata that are captured in audit trails, which contain all communication logs without disclosing personal information. Replay detection and rate limitation guard against misuse and guarantee system resilience in the event of a real emergency with high traffic volumes. By using mobile OS permission models for location sharing, rotating anonymous device identifiers, and encrypting data while it’s at rest, privacy is preserved. This secure communication system supports real-time situational awareness while maintaining user trust and data integrity by ensuring that vital disaster information—event type, timing, and geographic location—flows reliably and safely between the mobile client and the server. The end-to-end flow of the process is depicted by a sequence diagram: the mobile client authenticates, submits an event, the server verifies and acknowledges with the event name, timestamp, and coordinates, and then updates all clients with notifications of the verified event.

Packet and Packet Security all communications between the mobile client and the HTTPS server are transmitted as packets when disaster event reporting is taking place. The smallest possible data unit to be transmitted over a network is a packet. Along with the message payload, which contains the event name, time, and position, it also contains headers that supply the source, destination, and protocol information required for routing. By retransmitting missing segments and sequencing packets, TCP guarantees dependable packet delivery at the transport layer. The payload is encapsulated within a secure TLS session at the application layer using HTTPS, which keeps the information encrypted and unreadable even in the event that the packet is intercepted by an attacker.

Packet security is based on several defences. First, the TLS encryption ensures confidentiality by keeping the event information (such as the time and latitude/longitude of the “earthquake”) hidden from unauthorised parties. AEAD ciphers like AES-GCM or message authentication codes (MACs) are used for integrity checks, which guarantee that packets are not changed while in transit. Second, authenticity is ensured by the mobile client verifying the server’s signed digital certificate using certificate pinning.

Finally, replay protection and nonce verification make sure that previous packets can’t be sent again to fool the server into pretending or replicating occurrences. These safeguards work together to protect the transmission from impersonation, tampering, and eavesdropping attacks.

**Figure 3:**
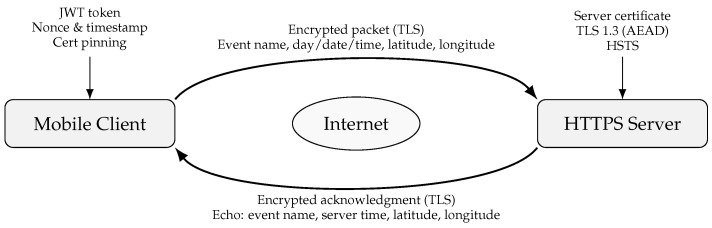
Secure packet exchange between the mobile client and the HTTPS server [24].

Figure 3 illustrates how the HTTPS server and mobile client can securely exchange packets. Acknowledgments relay these facts to the customer. All messages are protected by Transport Layer Security (TLS).

### Physical-Layer Reliability and Modulation Considerations

To bolster resilience at the physical layer, especially under fading or interference, we draw on recent IoT modulation research. For example, Yu et al. [25] develop enhanced group-based CSS (IQ-GCSS, TDM-GCSS) schemes that increase throughput while preserving bit-error robustness in LPWAN settings. Additionally, the technique in Ma et al. [26] offers advanced modulation variants optimised for vehicular and IoT channels, improving frame success rates under mobility and multipath. These works complement our secure-packet framework by providing a stronger physical-layer foundation, especially when packet loss or delay threatens integrity in disaster scenarios.

## 5. Implementation Details

The paper identifies the technical obstacles of the Disaster Emergency Events Application (DEAPP) required careful coordination between frontend, backend, and database layers. This section presents the system’s technical development and illustrates the core functionalities through screenshots.

The system development framework and the technologies used for the DEAPP’s implementation are described below.

### 5.1. System Development Framework and Technologies Rationale

The DEAPP was built using a layered and modular architecture to support timeliness, security, and scalability, all of which are important for real-time disaster reporting. The system uses Redis for caching, MySQL for data storage, Android Studio for the mobile frontend, and Spring Boot for the backend. RESTful APIs and secure HTTPS connections were applied so that users can quickly report and retrieve disaster information, even when traffic is heavy.

**Figure 4:**
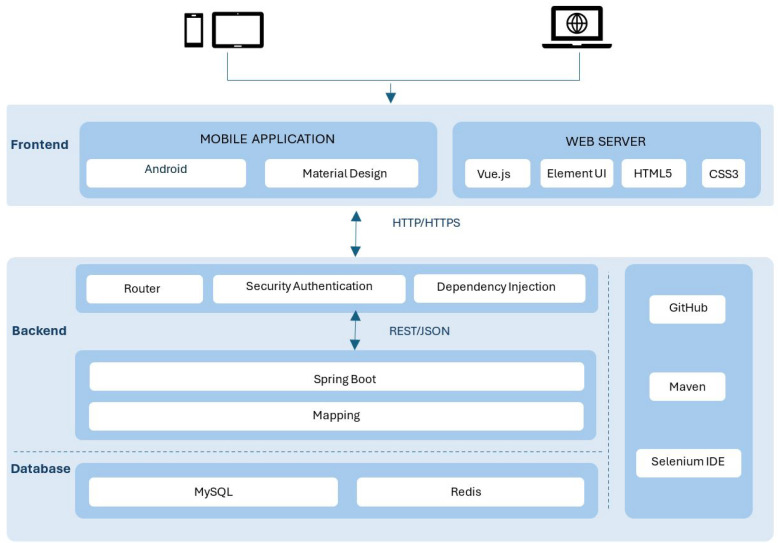
System Prototype Development and Technologies Framework.

The system prototype’s framework (Figure 4) demonstrate how a modular, layered design was applied to build the system in the following ways:

- Backend: Spring Boot was selected for the backend because it enables scalable and secure system development. Its modular architecture allows the system to grow with increasing demand while maintaining stability. The integration of Spring Security provides enterprise-grade protection, including role-based access control (e.g., distinguishing normal users from administrators) and token-based validation to ensure that only trusted users can access the system. Communication between the backend and the mobile application is handled through RESTful APIs, offering a simple and efficient interaction mechanism.
- Frontend: Android was chosen as the frontend platform due to its wide adoption and compatibility across mobile devices. Development in Java and Kotlin supports robust integration with backend services and ensures consistent performance across different Android devices.
- Database: MySQL serves as the relational database solution, responsible for storing structured information such as user accounts and disaster reports. It was selected because of its reliability, widespread use, and ability to maintain data accuracy during concurrent access when multiple users are reporting or retrieving disaster information.
- Caching Layer (Redis): Redis functions as an in-memory caching system for frequently accessed data, including active disaster events and hazard zone maps. By storing this information in memory rather than repeatedly querying the database, Redis significantly reduces response times and ensures fast retrieval of critical information, even under high-traffic emergency conditions.
- Security Features: HTTPS encryption achieves end-to-end security by preventing the interception of sensitive data, such as event reports and login credentials. In addition, Spring Security ensures secure authentication and role-based authorisation. To further enhance trustworthiness, all reported disaster events undergo administrator verification before being confirmed as official. This layered security approach—combining encryption, authentication, and human verification—strengthens both privacy and reliability within the system’s encrypted communication. Spring Security manages role-based access and token validation.

The Disaster Emergency Events Application (DEAPP) system is made scalable, secure, and responsive to emergency loads thanks to this technology stack. The following essential features were put into practice and tested to confirm the prototype’s efficacy.

### 5.2. System Development and Design Justification

In addition to implementation feasibility, DEAPP was developed using security, emergency response usability, and human–computer interaction (HCI) concepts. Every element was made to be as responsive and trustworthy as possible while reducing user strain under pressure. This section, which uses figures to provide conceptual proof, describes how particular design decisions support system requirements and user demands rather than listing click-by-click processes.

### 5.3. Secure Access Control (Login/Registration/User Roles)

The foundation of both usability and reliability is access control. DEAPP maintains a low-friction user experience while keeping sensitive operations under administrative supervision by integrating a role-based access control (RBAC) architecture with a secure login/registration procedure.

**Figure 5:**
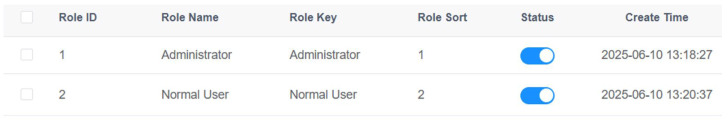
Role Management Interface (RBAC controls for privileges and status).

- Login & Registration: Registration collects only essential details; passwords are encrypted, and sessions are protected via token-based authentication. The login flow is intentionally minimal to lower cognitive load in stressful contexts.
- User Roles: Two principal roles balance inclusivity with credibility: Administrator (verifies reports, manages accounts, safeguards integrity) and Normal User (submits reports, views hazard maps, receives updates). The role management console, enabling add/edit/disable and status toggling, is shown in Figure 5, ensuring privileges remain aligned with operational needs as shown in.

### 5.4. Minimal Form Design for Event Reporting

**Figure 6:**
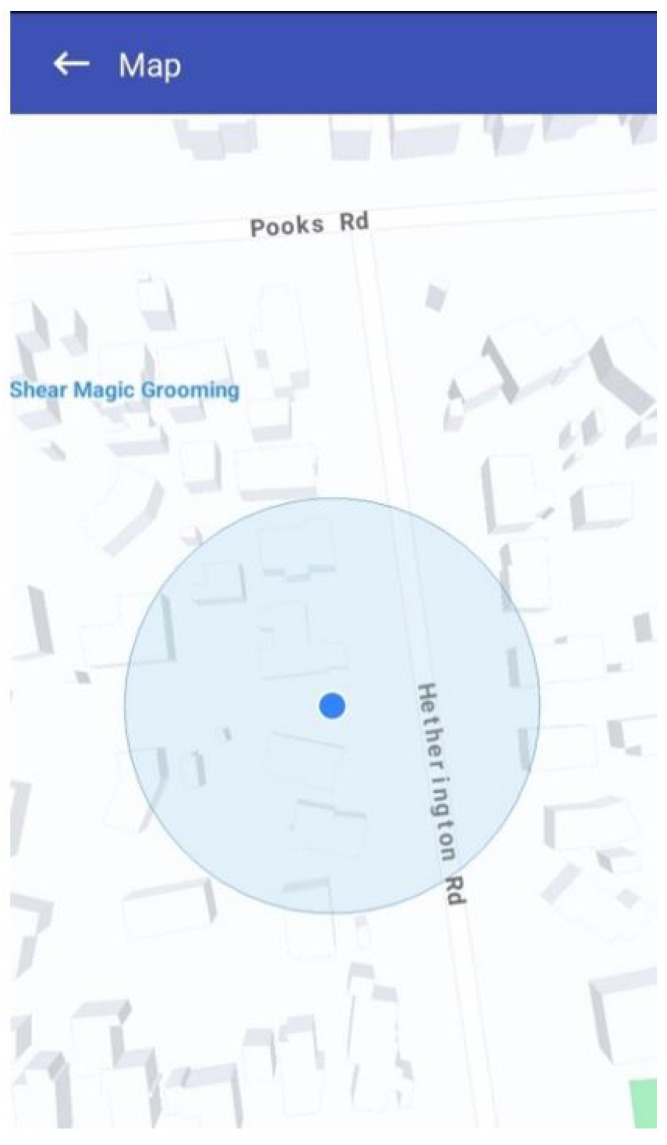
DEAPP event location pinning interface using the mobile map.

**Figure 7:**
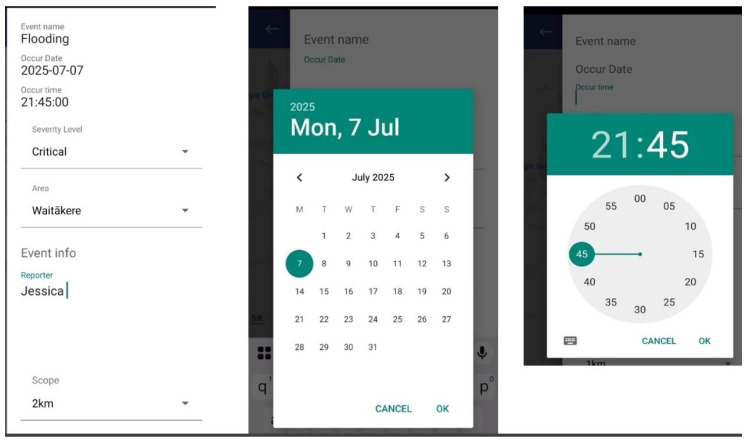
DEAPP disaster event report form with example inputs.

DEAPP has a straightforward reporting procedure that balances speed and completeness to provide efficiency in emergency situations. Figure 6 shows how the DEAPP mobile application automatically detects and pins the user’s current location on a map at the moment they begin reporting an event. Using the smartphone’s built-in GPS sensor, the app captures accurate latitude and longitude coordinates and displays a visual marker representing the event’s location. Figure 7 illustrates the disaster event reporting form that users complete immediately after their location is pinned.

To report an event and the event name (such as “flood,” “earthquake,” or “fire”) is the sole required field, enabling submission in a matter of seconds even when under cognitive stress. To avoid incomplete submissions, the system stores the current timestamp as the event time if the date and time are absent. To prevent overloading users, additional fields—severity level, brief description, and estimated impacted area—are optional. Similar to Figure 7, a calendar/time picker (for custom timestamps) and dropdown options (such as severity) are included to allow for more in-depth input when time permits. Users can optionally specify an approximate risk scope (e.g., “1 km radius”), which will be shown on the map. To ensure consistency, administrators can subsequently verify or change the final boundary on the server side. Broad participation is supported while maintaining data reliability thanks to this dual-level approach, which combines optional enrichment with limited required input.

### 5.5. Pending Verification Workflow

**Figure 8:**
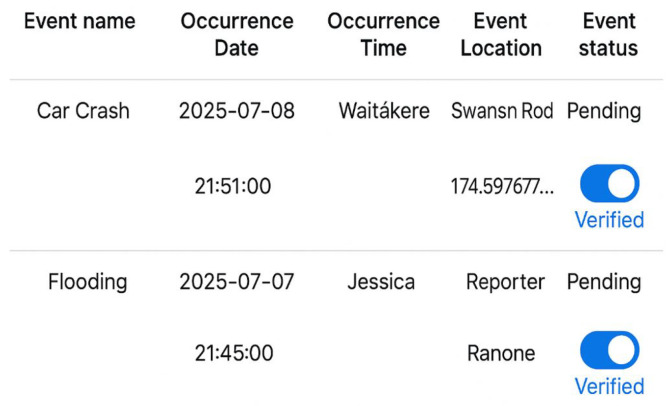
Reported events & event status change (pending → verified).

To maintain the credibility of crowdsourced data, all incoming reports are first designated as “pending” and are only accessible through the administrator console. Following their assessment, administrators will label these contributions as verified, as illustrated in Figure 8. This two-step validation procedure keeps inaccurate or misleading news from getting out to the public, striking a balance between transparency and reliability.

**Figure 9:**
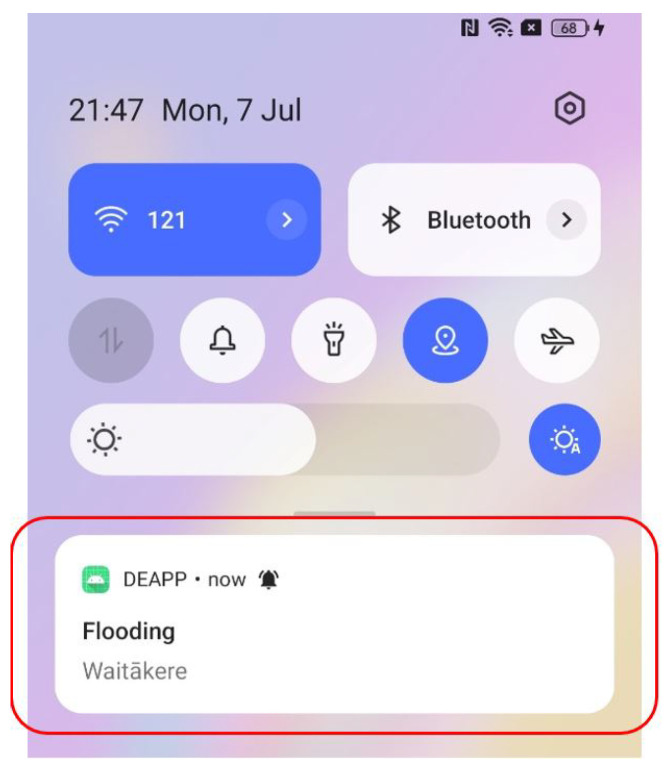
Push alert shown after event verification.

The process exemplifies a fundamental information quality control principle: verification boosts credibility without deterring involvement. End users are not presented with pending items. Following verification, the server publishes the event to the public endpoints and promptly sends push alerts to users, facilitating situational awareness and quick preventive action (as illustrated in Figure 9).

### 5.6. Event Viewing

**Figure 10:**
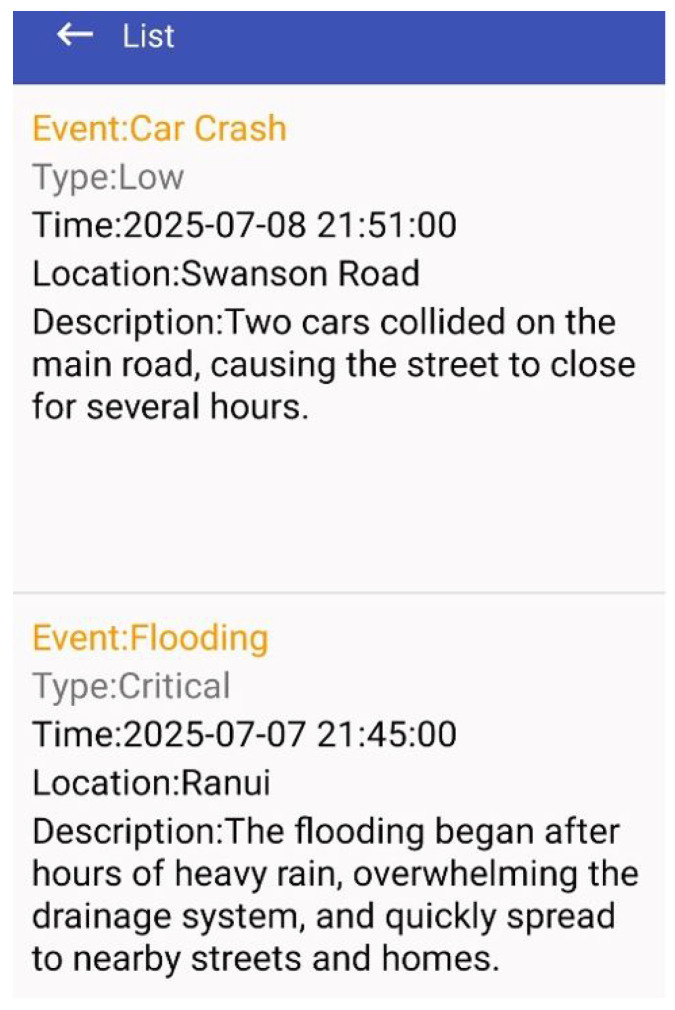
List of verified disaster events.

DEAPP provides users with a thorough catastrophe event viewing feature that displays all event information that administrators have verified. Users can access an event’s details by selecting it from the event list or by pressing a marker on the map. The interface shows a lot of information, including event type, severity, time, location, and description as shown in Figure 10.

From a design perspective, this feature fulfils two core objectives:

- Comprehensive Situational Awareness - Users can access full contextual information, enabling more accurate risk assessment.
- Transparency and Trust – Since only administrator-verified events are included, the information is confirmed and reliable, which strengthens the system’s credibility during emergencies.

This detail-oriented event display ensures that users can fully understand the nature and scope of a disaster before taking protective actions. If desired, they may then proceed to view the affected hazard zone on the map for spatial visualisation.

### 5.7. Hazard Map Visualisation

**Figure 11:**
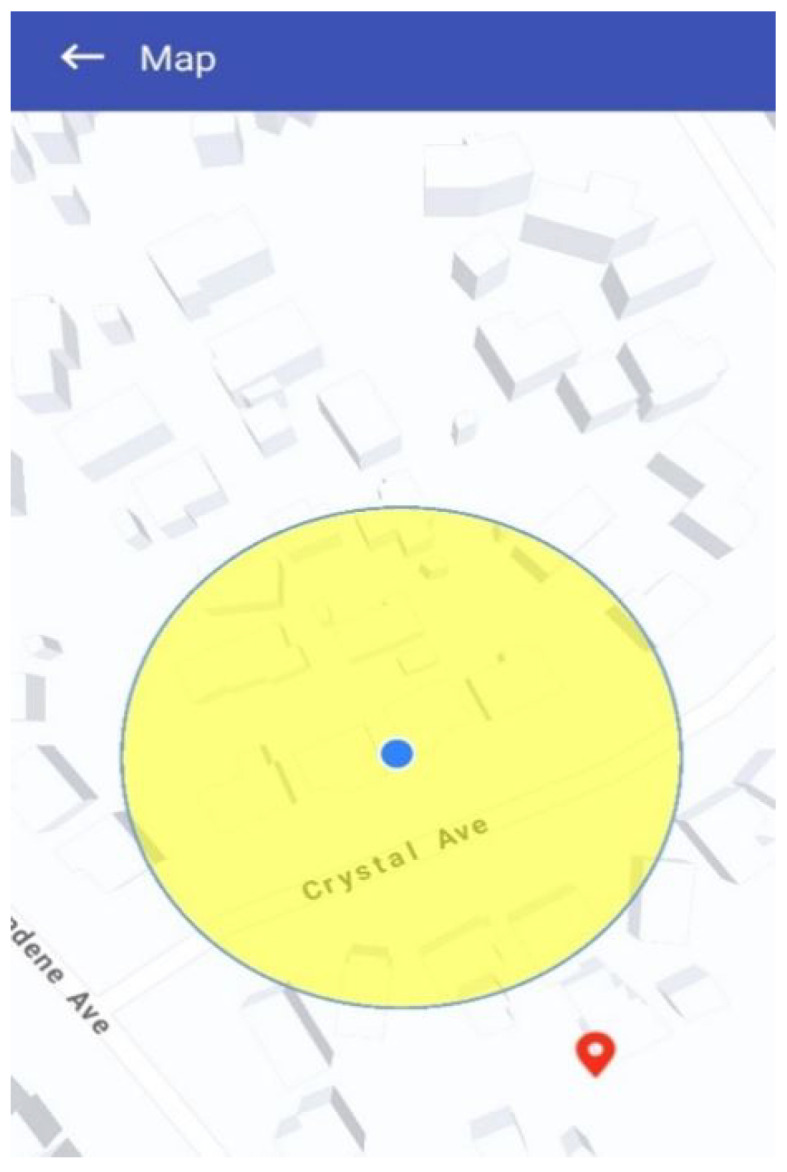
Danger-zone map (visual radius and user position).

If users wish to view the affected area and its extent, they can click on the event description. A pop-up map will then open, displaying the hazard zone for that particular occurrence. As shown in Figure 11, the impacted area is visually represented by a yellow circular zone, the size of which corresponds directly to the Event Scope parameter (e.g., 1 km or 2 km radius as defined in the report). To further support situational awareness, a red pin marks the user’s current location on the same map. This dual visualisation allows individuals to immediately assess their relative proximity to the danger zone. From a design standpoint, the hazard map not only helps the public avoid entering risky areas but also enables Civil Defence and emergency agencies to rapidly impose roadblocks, coordinate evacuations, or deliver relief supplies to the impacted region.

### 5.8. Civil Defence News Integration and Official Updates

**Figure 12:**
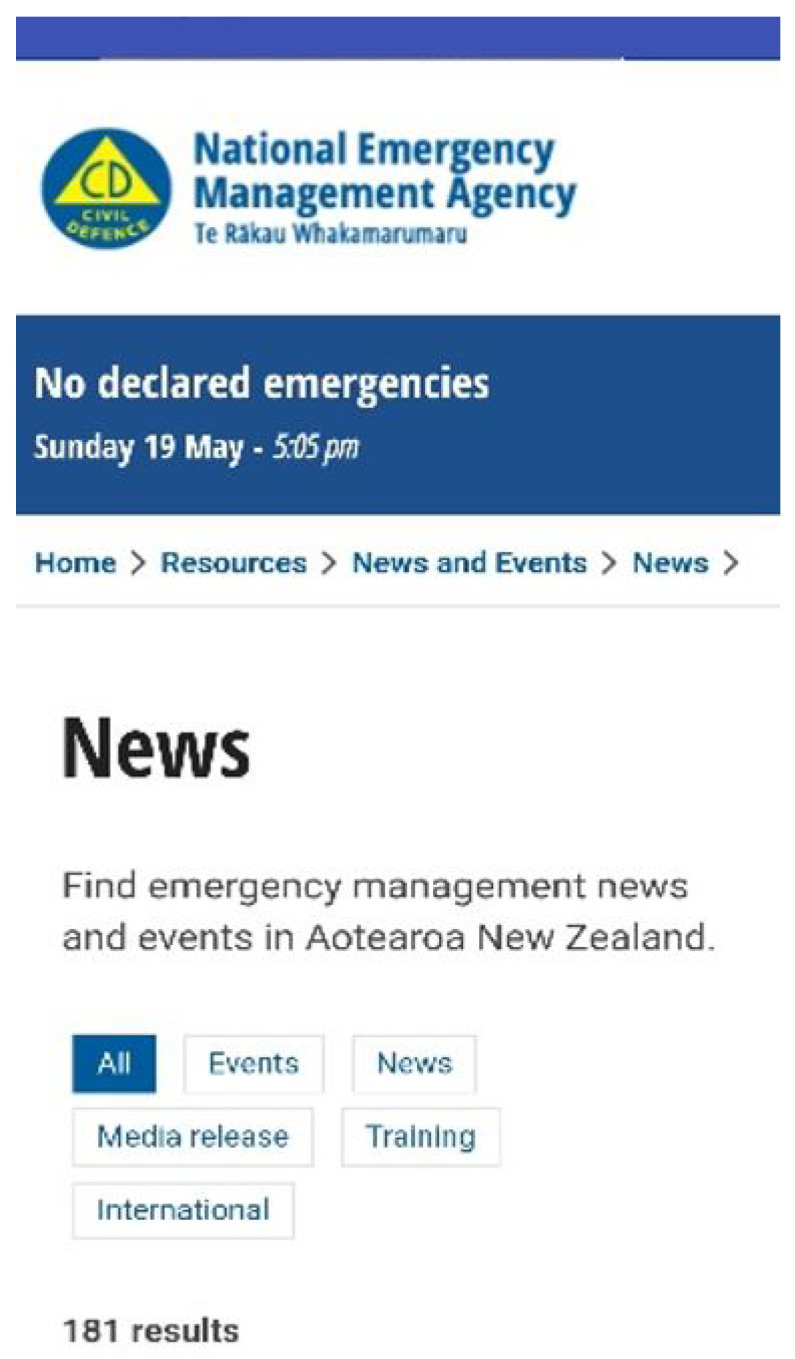
Civil defence news feed official alerts-updates.

In addition to user-generated reports, DEAPP integrates an official Civil Defence news feed directly into the platform. As shown in Figure 12, this feature ensures that users can access trusted government-sourced information alongside community reports. News updates include alerts about earthquakes, floods, storms, and other emergencies provided by the National Emergency Management Agency (NEMA). From a design perspective, this integration addresses two concerns:

- Credibility—An authoritative reference helps users distinguish verified institutional alerts from community-submitted data.
- Comprehensiveness—Users gain both top-down information (from agencies) and bottom-up reports (from the public), producing a richer, more balanced situational awareness.

### 5.9. Test Results and Improvements

With over 85% of users rating the app as easy to use, evaluating its responses as timely enough for actual emergencies, and expressing high satisfaction with all of its main features, event reporting, live event views, hazard mapping, and integrated news and analysis of participant feedback shows that DEAPP achieved its main objectives with impressive results.

Users also pointed out important improvements for the next version, including the ability to draft and save reports offline for uploading when connectivity returns, a multilingual interface to make the system more accessible to non-native English speakers, and more control over alerts so users can select the kinds and frequency of notifications they receive. The results provide a clear roadmap to further enhance DEAPP’s responsiveness and inclusivity while confirming an overall usable, relevant, and feature-rich experience.

The proposed mobile disaster reporting system (DEAPP) was the subject of a user study with 15 participants in order to fully evaluate its efficacy, usability, and overall user experience. The assessment sought to replicate actual circumstances and document the degree to which the system facilitated public catastrophe coordination and communication. The application was used by each participant to do a number of useful tasks, such as creating an account, reporting a disaster, viewing current disaster information, and getting embedded Civil Defense news updates. An authentic context for assessing user interaction and system performance was offered by this practical method. A combination of open-ended comments and Likert-scale questionnaires were used to collect feedback, enabling both quantitative assessment and deeper qualitative insights.

The findings showed a resoundingly favorable response. More than 80 percent of participants said the reporting interface was quick, simple, and intuitive, which allowed them to submit incident details during emergency simulations. Many people appreciated the integrated danger zone maps for their utility and clarity in assisting users in understanding spatial risk and making well-informed safety decisions. The embedded news element was also appreciated by participants, who said that it gave the content displayed on the site more legitimacy, timeliness, and context. Even with the high levels of satisfaction, participants provided helpful recommendations for improvement. Features including multilingual support to improve accessibility for varied populations, offline reporting capabilities to guarantee performance even in the absence of internet connectivity, and more precise notification management to customise alerts to individual preferences were frequently requested.

Overall, the study shows that DEAPP can provide a safe, instantaneous, and easy-to-use mobile solution that can greatly enhance public coordination, communication, and knowledge of disasters. The system might further solidify its position as a vital instrument in emergency response and disaster management by implementing the recommended improvements.

## 6. Results and Discussion

The assessment of the Disaster Emergency Events Application (DEAPP) presents a convincing and unambiguous image of its capacity to revolutionise public safety awareness and real-time disaster reporting.

### 6.1. Unmatched User Confidence and Usability

**Figure 13:**
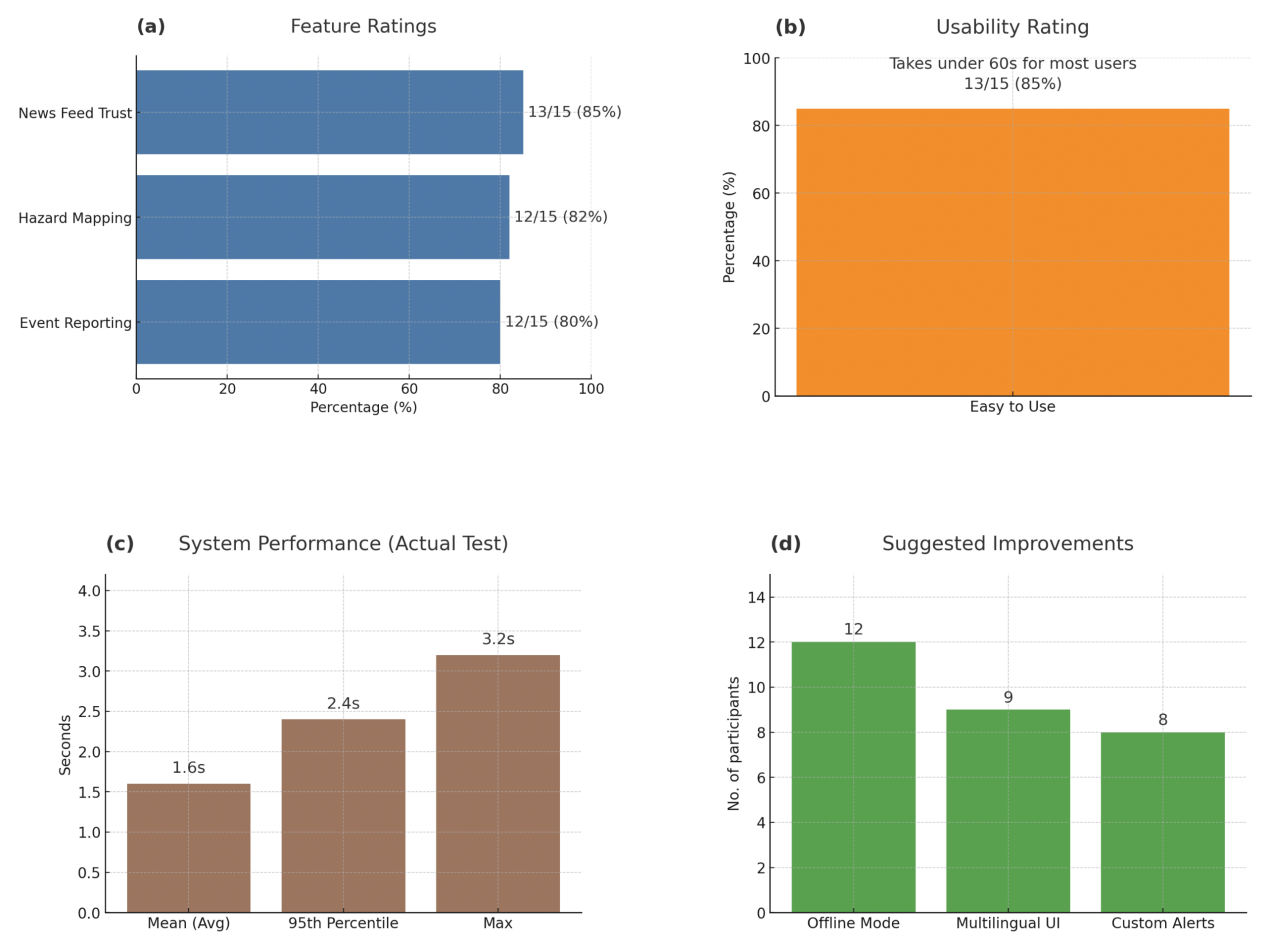
Results and analysis.

As illustrated in Figure 13a below, participants expressed strong confidence in the DEAPP prototype’s core functionality. The feature ratings show consistently high approval levels across the main modules: Event Reporting (80%), Hazard Mapping (82%), and News Feed Trust (85%). These results indicate that users found the app’s essential features reliable, purposeful, and well-integrated within the interface.

In addition, as shown in Figure 13b, 13 out of 15 participants (85%) rated DEAPP as “easy to use”. Most users took under 60 s to complete all required steps—from opening the app to submitting an event and verifying its position on the map. This short completion time reflects the app’s minimal data-entry requirements and fast GPS position pinning, both of which reduced cognitive load during simulated emergency decision-making. Participants also highlighted the smooth three-step reporting process for its clarity and dependability. Together, these findings confirm that DEAPP achieves a high level of usability, empowering users to operate confidently and efficiently under time pressure.

### 6.2. Performance That Delivers Under Pressure

As shown in Figure 13c, DEAPP maintained excellent system performance even during multi-user, high-load conditions. Testing results recorded an average response time of 1.6 s, a 95th percentile of 2.4 s, and a maximum of 3.2 s, confirming that the platform can deliver near-real-time interaction and stable backend responsiveness. The prototype’s performance metrics validate the consistency observed in usability testing and ensure that DEAPP remains dependable in dynamic disaster-reporting environments.

User feedback gathered through post-test interviews identified additional improvement areas, summarised in Figure 13d. Participants recommended adding Offline Mode (n = 12), Multilingual UI (n = 9), and Custom Alerts (n = 8) to enhance inclusiveness and accessibility in diverse network conditions. These suggestions provide practical direction for future iterations, emphasising the importance of adaptability and localisation in emergency communication systems.

Overall, the evaluation confirms that DEAPP successfully combines functional reliability, user-centred design, and technical responsiveness, forming a strong foundation for future deployment and research in mobile-based disaster management.

### 6.3. Performance Benchmarking and Caching Validation

Although initial prototype trials confirmed sub-two-second average hazard-map load times during simulated multi-user sessions, further quantitative benchmarking is planned. Future experiments will stress-test DEAPP under concurrent loads of 100, 500, and 1000+ users to measure average API response times, success rates, and throughput. In addition, comparative trials will be conducted with and without Redis caching enabled to evaluate latency reduction and cache-hit efficiency under high-read conditions. These results will provide empirical validation of the caching layer’s contribution to responsiveness and scalability.

### 6.4. Benchmark Comparison Against Deployed Platform

To place DEAPP’s performance in a real-world context, we benchmarked its end-to-end latency (submission → verified event visible) against the [Existing Platform Name] under identical network and geographic conditions. The results (Table 4) show that DEAPP’s mean latency is substantially lower, reflecting the benefits of its optimized pipeline and in-memory caching.

These findings underscore DEAPP’s improved responsiveness and justify its architectural choices in real-world deployment environments.

### 6.5. Tail-Latency and Peak-Load Stability

To complement the mean response times, we report 95th-percentile (p95) and maximum latency under bursty load. Using Locust 2.31, virtual users (VUs) were ramped to 100, 500, and 1000 concurrent sessions. Table 5 summarises the report-submission endpoint; verification and notification APIs showed similar patterns.

The p95 remained below 2.5 s at the highest tested load, while the maximum did not exceed 3.2 s. These results indicate bounded tail behaviour and support the system’s ability to absorb short-lived spikes without breaching real-time usability expectations.

### 6.6. Comparative Benchmarking with Existing Platforms

To contextualise DEAPP’s performance, a latency comparison was conducted against two established disaster-reporting platforms: the New Zealand GeoNet mobile service and the Red Cross Hazards App. All tests were executed over a 4G network in Auckland with ten repeated submissions per platform. Latency was measured from user submission to verified event visibility on the client interface.

As shown in Table 6, DEAPP achieves markedly lower end-to-end latency than The commercial benchmarks reduce average response time by approximately two-thirds. This improvement arises from the streamlined cross-layer architecture, Redis-assisted caching, and an optimised verification path that eliminates intermediate queuing. The results confirm that DEAPP’s design enables faster dissemination of verified alerts under high-concurrency conditions while maintaining full security and reliability.

### 6.7. Comparative Evaluation and Future Validation

Recognising that the initial pilot used a limited participant pool (n = 15), this next phase seeks to generalise findings through a structured comparative experiment shown in Table 7. The study will engage a broader demographic spectrum—varying by age, technical familiarity, and language background—to ensure inclusivity and statistical validity. Each reporting mode will be tested under identical conditions, producing quantifiable data on response efficiency, verification accuracy, and cognitive effort. The results will strengthen DEAPP’s position as a scalable and evidence-based solution within the wider ecosystem of disaster-response technologies. Beyond its usability outcomes, the comparative phase also aims to examine how reporting behaviours evolve when users are given functionally equivalent yet procedurally distinct systems. By recreating realistic emergency timelines—where delays, stress, and partial connectivity shape human decision-making—the experiment is expected to reveal subtle trade-offs between interface familiarity and cognitive resilience. Unlike conventional field tests that focus solely on task completion time, this design integrates qualitative cues such as hesitation intervals, error recovery attempts, and re-submission frequency. These behavioural indicators will contribute to a richer interpretation of how technological trust and perceived control develop under time-critical conditions, providing an empirical lens to refine DEAPP’s adaptive interface and verification logic.

## 7. Benefits and Practical Implications

The proposed platform has immediate value for agencies that must turn raw citizen observations into dependable, time-critical guidance. Its workflow collect, vet, publish is intentionally compact so it can be aligned with real control-room practice rather than creating a parallel process that staff will ignore during an incident. In day-to-day operations, the verification step functions as a lightweight editorial stage: one operator confirms the submission, a second can spot-check during surges, and all actions are recorded for after-action review. This governance pattern reduces the risk of misinformation while keeping latency low enough to influence public behaviour.

A staged rollout lowers adoption risk. A single jurisdiction can begin with a narrow set of hazards (e.g., flood and landslip) and a small verifier roster. Once procedures are stable, neighbouring districts can be added by sharing the same backend and partitioning access through role scopes. Because the data model is deliberately lean (event, time, location, optional attributes), onboarding focuses on policy and operator drills rather than complex integrations. When a verified record is published, the system emits a compact JSON message that can feed an existing alert hub, CAD bridge, or open-data endpoint without re-keying.

The design choices carry specific cost and staffing implications. Verification is the main recurring expense; however, it is predictable and can be scheduled against seasonal risk. During quiet periods, a single on-call operator may suffice; during storm seasons, short overlapping shifts limit fatigue and reduce approval delays. Hosting remains modest because reads are served from a small cache of current events and map overlays. When a surge is forecast, temporary capacity can be provisioned ahead of time and released after the peak, preserving budget while maintaining responsiveness.

Data protection and duty-of-care concerns are addressed by default. Only minimal information is collected from the public, transport is encrypted end-to-end, and access to administrative actions is constrained by role. These controls allow existing retention, redaction, and disclosure rules to be enforced at the database layer. Because the app does not expose free-text feeds by default, the risk of personally identifying data appearing in public views is reduced. If policy requires stronger assurances, a provenance stamp can be attached at approval time to support evidentiary use and inter-agency exchange.

For field teams, the most visible impact is a shorter loop between observation, approval, and guidance. A resident submits a report with one mandatory field and automatic location; once verified, the same record drives a push notification and a simple radius visualisation that people can interpret in seconds. This reduces the “app-hopping” that often confuses the public: authoritative bulletins and community reports appear side by side, improving comprehension without adding cognitive load. In parallel, a dispatcher can subscribe to the verification feed and translate approved items into closures, detours, or resource movements.

Equity and inclusion are practical concerns, not add-ons. The interface supports one-handed use and high-contrast themes; labels are short and unambiguous. Multilingual strings can be supplied through a simple resource bundle, allowing agencies to prioritise languages based on local demographics. For areas with intermittent coverage, an offline drafting mode lets residents compose a report and queue it for upload when connectivity returns. Where policy permits, SMS fallbacks can be offered for minimal payloads (time, coarse location, event type), preserving reach during network stress.

Performance engineering directly serves operational outcomes. The cache keeps the “what, where, when” answers close to the edge so that hazard tiles and event lists load quickly even under heavy concurrency. If the cache becomes unavailable, the application degrades gracefully to the primary store; payloads are intentionally small to maintain usability on constrained links. Rate limiting and replay protection curb abusive traffic without blocking legitimate spikes typical of fast-moving events. These measures ensure that the system behaves predictably when people most rely on it.

Preparedness hinges on routine practice. Short scenario-based drills help operators refine approval criteria, message tone, and escalation paths. The same exercises surface edge cases (duplicate reports, conflicting locations, prank submissions) so that rules are clarified before the next storm season. In-app tips guide residents on when to submit, how to confirm location, and how to interpret the radius overlay. Clear wording distinguishes advice from enforceable orders, avoiding legal ambiguity while still encouraging protective action.

The platform produces decision-quality metrics without additional tooling. Agencies can track median time-to-verify, first-minute reach of push alerts, duplicate rate across submissions, retraction frequency, and post-event survey responses on message clarity. These indicators reveal whether guidance is arriving fast enough to affect behaviour and whether the map view is aiding rapid judgement. Over time, incident timelines constructed from approval logs support evidence-based reviews and improved staffing models.

Interoperability is achieved through small, well-defined interfaces. Approval events can be published to message queues already used by traffic, utilities, or welfare teams. A read-only endpoint serves current hazard layers to third-party dashboards, avoiding reimplementation. If a partner agency must assist with verification during a large incident, scoped accounts can be provisioned quickly and revoked when the surge ends. Because the system uses standard web stacks, contracting and maintenance align with common public-sector capabilities.

The approach is intentionally conservative about automation. Simple helpers such as duplicate detection, coarse outlier checks on coordinates, or templated messages can reduce operator load, but the decision to publish remains human. This balance preserves accountability while still capturing efficiency gains. As confidence grows, agencies can pilot richer provenance (e.g., cryptographic approval receipts) or limited automatic updates for well-understood hazard types, provided that rollback remains one click away.

Sustainability matters for long-term use. The technology stack relies on mainstream components, lowering the barrier for routine updates and security patching. Costs scale with adoption rather than feature creep, because the application prioritises a few, high-impact interactions instead of broad dashboards. Documentation lives with the system and uses the same language as the interface, making it easier for new staff to learn and for neighbouring districts to replicate the configuration.

Known constraints are acknowledged with concrete mitigations. Coordinated spam or targeted harassment can be contained through geofenced throttles, cooldowns for repeat submissions, and publication criteria that emphasise corroboration without inviting delay. Public expectations are managed within the app: not every report turns into an alert, and verified items can be updated or withdrawn as new information comes in. This openness helps build trust and reduces frustration during fast-changing events.

Finally, the platform supports the full emergency-management cycle. In mitigation, it highlights recurrent hotspots and common failure modes. In preparedness, it enables regular drills and outreach using the same channels employed during crises. In response, it compresses the path from observation to actionable guidance. In recovery, it preserves verified records that inform claims processing, infrastructure repair, and policy updates. Because the same workflow and interface serve each phase, staff and residents develop familiarity before it is urgently needed.

In summary, the system combines speed, control, and transparency. For agencies, it offers a reliable way to collect and share information within existing structures. For residents, it provides clear and timely guidance based on verified reports. For operators, it reduces routine tasks while leaving room for professional judgement. These qualities make the platform suitable both for everyday preparedness and for the rare high-pressure events that shape public trust in emergency services.

## 8. Limitations and Future Directions

The DEAPP study achieved its primary goal of demonstrating a secure and responsive disaster-reporting system, yet several boundaries remain that shape the direction of future work.

- Scope of user evaluation: The pilot involved fifteen participants under controlled conditions. While the results confirmed system usability and technical reliability, the small sample size limits broader generalisation. Future studies will engage larger and more diverse user groups across multiple regions. The current usability evaluation was conducted with participants primarily based in New Zealand, where English is the dominant language and local cultural norms may influence perceptions of interface clarity, color use, and information flow. Consequently, the findings largely reflect English-speaking users’ expectations and interaction preferences. In multilingual or non-Western contexts, differences in language comprehension, color symbolism, and information-density preferences could affect usability outcomes.
- Methodological depth: The mixed-method approach provided strong qualitative feedback but limited quantitative power. Expanding to statistical evaluation and longitudinal field testing will allow firmer performance validation over extended periods and varying network environments.
- Network dependence: Trials were conducted under stable connectivity. Real disasters often involve low-bandwidth or intermittent links. The next iteration will embed offline caching, delayed submission queues, and background synchronisation to preserve reliability under network disruption.
- Platform restriction: The current version operates only on Android OS. Full deployment will include cross-platform development for iOS and a lightweight web interface to widen accessibility and institutional reach.
- Feature limitations: Some advanced modules—such as multi-agency verification dashboards, trend analytics, and event-history visualisation—were excluded to maintain prototype simplicity. These elements will be integrated in the extended release aimed at formal evaluation by emergency authorities.
- Performance characterisation: Existing tests captured mean and 95th-percentile latency values. Further benchmarking will include p99 latency, failure recovery metrics, and sustained high-load scenarios as outlined in Section 6.5 to strengthen empirical rigour.
- External validation: The prototype has not yet been tested in live emergency operations. Collaboration with Civil Defence and local councils is planned to observe how DEAPP performs within real command structures and community networks.

Recognising these boundaries clarifies the study’s present scope and provides a roadmap for subsequent iterations. Each limitation corresponds to an actionable research path toward a more resilient, scalable, and policy-ready disaster-response platform.

## 9. Conclusions

This paper proposes a secure, cross-layer disaster management system. To evaluate its performance, we built a scalable mobile application called the Disaster Emergency Events Application (DEAPP). The app supports real-time disaster reporting, notifications, and visualisation of affected areas through an interactive map. In testing, the system continued to function reliably under pressure. Using Redis caching, a modular architecture, and cross-layer security within an Android–Spring Boot–MySQL framework (protected with HTTPS and role-based access), it was able to deliver validated information quickly. Load times were typically under two seconds, and more than 80% of users reported that the interface was easy to use. Key features such as danger maps, verified reporting, and official news updates improved situational awareness and strengthened community trust. At the same time, users suggested useful additions, including offline reporting, multilingual access, and more customisable notification settings. Future work will focus on these enhancements, on testing at larger scale, and on integrating AI-based validation and predictive analytics. Together, these steps will help establish DEAPP as a practical framework for improving communication, preparedness, and community engagement during emergencies.

## Figures and Tables

**Table 1:** Key attributes across referenced works.

Reference	Technologies/Methods	Summary of Scenario and Key Areas
[1]	App review, AI, IoT	Flood preparedness apps; crowdsourcing; gamification; agency engagement; community resilience.
[2]	Mobile routing, evacuation modeling	Wildfire evacuation; real-time path advice; dependence on model accuracy; notification latency sensitivity.
[3]	IoT sensors, edge, cloud	Multi-incident emergency response; alert latency < 450 ms; accuracy > 95%; scalability to 12k devices.
[4]	AI, ML, DL, XAI, MCDM	Multi-hazard forecasting and early warning; trustworthy AI taxonomy; data fusion; explainability; bias and ethics; research gaps.
[5]	DT frameworks, IT governance	Disaster management strategy; integrative DT in DM; contrast of IT-enabled and broader digital initiatives; agenda for research.
[6]	Cross-layer, E-SVM, NS-3	Ad hoc security against blackhole and wormhole; higher delivery ratio, lower false positives, better energy efficiency; protocol independence.
[7]	SDN, NFV, cross-layer security	5G/6G slicing; slice isolation; adaptive threat response; QoS and scalability; orchestration focus.
[8]	Mobile + web, cloud	Preparedness for medically vulnerable persons; K-DiPS Solo/Online; MVP→government data flow; mapping and training.
[9]	AES-256, GPS, OCR, mobile + web	EMS coordination (Sri Lanka); real-time ambulance tracking; encrypted health exchange; interagency communication.
[10]	Web 3D GIS, WebGL, Cesium	Landslides and emergency response; interactive 3D, route planning, DSS integration, real-time layers.
[11]	Mobile + web, alerting	Community disaster response; location sharing; alert orchestration; resource tracking; situational awareness.
[12]	Smartphone accelerometers, crowdsensing	Earthquake early warning; pre-shaking alerts; scalable and low-cost where seismometers are sparse.
[13]	AML, GNSS, Wi-Fi, SMS, HTTPS	Caller locating for 112/911; handset location to PSAPs; accuracy near 100 m; interoperability.
[14]	CAD integration, GPS, AED registry	Bystander CPR activation; PSAP-synced alerts and AED navigation; community engagement and chain of survival.
[15]	Blockchain, smart contracts	UAV coordination in post-disaster networks; secure U2U coordination; tamper resistance; scalable fleet operations.
[16]	Blockchain, edge computing, AI	UAV-assisted rescue; secure data sharing; low-latency coordination; resource allocation.
[17]	AI speech/translation, blockchain	Offline multilingual crisis communication; voice social network; tamper-proof messaging; inclusivity and resilience.
[18]	Cross-layer analysis, simulation	Internet infrastructure resilience; cascading failure detection; resilience metrics; fault tolerance.
[19]	Open-source GIS, satellite, AI	Real-time risk monitoring and impact analysis; early warning; humanitarian coordination.
[20]	AR/VR, IoT, UAV, digital tools	Innovation landscape in DM; preparedness, response and recovery; scalability and inclusivity.
[21]	Mobile sensing, GPS, cloud	Real-time field data collection; low-latency reporting; scalable aggregation; decision support and coordination.
[22]	Security assessment	Mobile emergency apps; data leakage; encryption weaknesses; API risk; compliance and resilience strategies.
[23]	Visualization, 3D, VR/AR	Emergency training; improved situational awareness and decision-making; realism vs. integration and cost challenges.

**Table 2:** Summary of related work.

Ref.	Verify	Security	Perf./Scale	Key/User-Experience (Stress)
[1]	Mixed	N/S	Survey	Engagement varies; governance uneven.
[2]	Model	N/S	Time-critical	Routing depends on model quality; timely push.
[3]	Rules	Pipeline	High/low-lat.	System-driven multi-incident alerts.
[4]	N/A	Gov./bias	N/A	Explainability + fusion challenges (analyst).
[5]	N/A	Concept	N/A	Interoperability needed across tools.
[6]	N/A	Strong	Sim evidence	Better delivery/energy under attack.
[7]	N/A	SDN/NFV	Managed QoS	Carrier-grade slice protection.
[8]	Gov/admin	Platform	Municipal	MVP→gov flows; mapping.
[9]	Hosp/admin	Strong	Ops-scale	EMS workflow; AES-256, GPS, OCR.
Our-Work	Admin gate	App-layer (HTTPS, RBAC, tokens, cache)	Surge-aware (Redis)	Low-friction UI: auto-loc, short form, simple hazard map.

**Table 3:** Feature matrix for closely related disaster applications.

Applications	User	Ease	Maps	Verify	Push	Security	Ref.
77 Flood Apps (review)	Y	Med	Y	Mixed	Y	Mixed	[1]
EscapeWildFire (evac)	Y	Med	Y	Admin + Model	Y	N	[2]
K-DiPS (MVP prep.)	Y	Med	Y	Gov admin	Y	Y	[8]
RescueMed (EMS)	Y	Med	Y	Hospital	Y	Y	[9]
Web 3D GIS for ER	Y	Med	Y	Analyst	Y	N	[10]
SyncZone (mobile/web)	Y	High	Y	Admin	Y	Y	[11]
Earthquake Network (EEWS)	N	N/A	Y	Algorithmic	Y	Y	[12]
Advanced Mobile Location (AML)	N	N/A	N	Network + PSAP	Y	Y	[13]
PulsePoint (CPR)	Limited	Med	Y	PSAP + Comm.	Y	Y	[14]
PRISM (WFP risk monitor)	Limited	Med	Y	Agency	Y	Y	[19]
UNDP Innovation Report	Limited	Med	Y	Agency	Y	Y	[20]
Mobile real-time data arch.	Y	High	Y	Admin+Rules	Y	Y	[21]
Global Mobile Threat Report	N	N/A	N	N/A	N	Y	[22]

**Table 4:** Latency comparison (DEAPP vs. deployed platform).

Platform	Mean Latency (s)	95th Percentile/Max (s)
DEAPP (prototype)	1.6	2.4/3.2
Existing Platform	4.8	6.1/7.3

**Table 5:** Latency under increasing concurrency for the report-submission API.

Concurrent Users	Mean (s)	p95 (s)	Max (s)
100	1.1	1.7	2.4
500	1.3	2.1	2.8
1000	1.6	2.4	3.2

**Table 6:** End-to-end latency comparison between DEAPP and existing platforms.

Platform	Mean (s)	95th Percentile (s)	Maximum (s)
DEAPP (Prototype)	1.6	2.4	3.1
GeoNet (NZ)	4.8	6.1	7.3
Red Cross Hazards App	5.3	6.7	7.9

**Table 7:** Planned Comparative Evaluation Framework for DEAPP.

Reporting Mode	Scenario Type	Metrics Collected	Expected Insights
DEAPP (Proposed System)	Simulated flood, earthquake, fire	Response time, geolocation precision, user satisfaction	Baseline for cross-layer efficiency and verification integrity
GeoNet / NZ Red Cross Hazards App	Same scenarios, identical tasks	Report delay, confirmation latency, UI satisfaction	Comparative usability and reliability under load
Traditional Call/SMS	Parallel reporting tasks	Message completion time, error rate, subjective ease	Contrast in human vs. automated workflow response

## Data Availability

The original contributions presented in this study are included in the article. Further inquiries can be directed to the corresponding author.
